# Efficacy of lumbar kinetic chain training for staged rehabilitation after percutaneous endoscopic lumbar discectomy

**DOI:** 10.1186/s12891-021-04674-y

**Published:** 2021-09-15

**Authors:** Zhen Lyu, Jinzhu Bai, Shizheng Chen, Jiesheng Liu, Wenlong Yu

**Affiliations:** 1 Department of Spine and Spinal Cord Surgery, Beijing Bo’ai Hospital, Rehabilitation Research Center, School of Rehabilitation, Capital Medical University, 10th JiaoMen North Road, Fengtai District, Beijing, China; 2 Department of Physical Therapy, Beijing Bo’ai Hospital,China Rehabilitation Research Center, School of Rehabilitation, Capital Medical University, Beijing, China

**Keywords:** Postoperative rehabilitation, Staged rehabilitation, Lumbar kinetic chain, Percutaneous endoscopic lumbar discectomy, Lumbar disc herniation

## Abstract

**Background:**

Percutaneous endoscopic lumbar discectomy (PELD) is a promising minimally invasive treatment for lumbar disc herniation (LDH). Postoperative rehabilitation can improve patient outcomes. Not only rehabilitation for surgical trauma but also rehabilitation for lumbar spine and lower kinetic chain dysfunction should be performed. The aims of this study were to investigate the efficacy of a lumbar kinetic chain training for staged rehabilitation after PELD for LDH.

**Methods:**

Fifty one LDH patients treated with PELD were studied. After surgery, patients underwent lumbar kinetic chain training for staged rehabilitation( staged group) or regular low back rehabilitation (regular group). The staged rehabilitation programme included three phases from 2 to 6, 7–12, and 13–24 weeks postoperatively, and different physical therapies were performed during these phases. The low back pain visual analogue scale (VAS), JOA score, ODI, SF-36, and cross-sectional area of the lumbar multifidus on MRI were assessed, and gait analysis was performed.

**Results:**

Twenty five patients in staged group and twenty six patients in regular group were included. There were no significant differences in age or sex between the two groups at baseline (*p* > 0.05). The VAS score decreased and the JOA and SF-36 scores increased in both groups from baseline to 6 weeks (*P* < 0.05). In the staged group, compared with the regular group, the VAS and ODI scores were lower and the JOA and SF-36 scores were higher at 6 weeks (*P* < 0.05); the VAS and ODI scores were lower and the SF-36 score was higher at 12 weeks (*P* < 0.05); the SF-36 score was higher at 24 weeks (*P* < 0.05); the cross-sectional area of the lumbar multifidus showed no differences at 12 weeks (*P* > 0.05); and the left-right support ratio of gait was higher at 24 weeks (*P* < 0.05).

**Conclusions:**

The staged rehabilitation programme for LDH after PELD promoted postoperative recovery, and the efficacy of lumbar kinetic chain training was higher than that of regular low back muscle exercise.

## Background

Minimally invasive surgeries of the lumbar spine have been developing rapidly in recent years. Many patients with lumbar disc herniation (LDH) have been effectively treated with percutaneous endoscopic lumbar discectomy (PELD). The procedure has been indicated to be minimally invasive and lead to a fast recovery [1, 2]. PELD removes the herniated nucleus pulposus and decompresses the nerve root. However, LDH causes not only nerve root compression but also lumbar spine and lower kinetic chain dysfunction [3, 4]. Postoperative rehabilitation should not only focus on lumbar surgical trauma but also ways to improve the efficacy of PELD, prevent recurrence of LDH, and further improve function.

Physical therapy and rehabilitation after lumbar decompression surgery are helpful for the functional recovery of patients [5–9]. However, due to the influence of factors such as the time at which postoperative rehabilitation is implemented, the number of cases studied and the design of the rehabilitation program implemented, the results of previous studies are inconsistent. Some studies have shown that early rehabilitation after lumbar disc surgery is not effective or cost-effective compared to no referral [10]. Referral for unstandardized municipal rehabilitation does not affect the time to return to work or the ability to work in patients recovering from surgery for LDH [11]. Therefore, it is particularly important to develop rehabilitation programmes according to the diagnosis and surgical procedure specific to the patient to help relieve pain and promote functional recovery.

PELD removes the herniated intervertebral disc, the corresponding ligamentum flavum and articular process. Physical therapy and rehabilitation are needed to achieve important postoperative goals such as increasing the strength of the low back muscles to compensate for surgical damage, preventing degeneration and instability of the adjacent segments, adjusting postural and kinetic models, correcting the mechanical imbalance of the pelvis and lower extremities and improving gait performance.

Disc degeneration is closely related to the paraspinal muscles [12–14]. LDH patients often have abnormal gait patterns due to pelvic and lower extremity mechanical imbalances [15, 16]. Core muscle training is commonly performed after lumbar surgery and can enhance lumbar stability. The McKenzie technique emphasizes strengthening the lumbar spine in extension and reducing disc pressure, and this standard technique is used for conservative LDH treatment and effectively improves symptoms [17, 18]. Postoperative rehabilitation programmes should be designed according to the characteristics of the different surgical recovery phases. We proposed staged rehabilitation and integrated lumbar-pelvic-leg kinetic chain training based on the McKenzie technique and core stability muscle exercise for LDH after PELD.

The aims of this study were to design a specific kinetic chain staged rehabilitation programme, compare it with regular low back muscle exercise and evaluate its efficacy.

## Materials and methods

### Patients

The study was approved by the ethics board of the Chinese Rehabilitation Research Center (register No. 2019-121-1), and all participants signed written informed consent forms. This study included 51 LDH patients treated with PELD in the Department of Spine Surgery of the Chinese Rehabilitation Research Center from January 2019 to June 2020. All patients underwent lateral-approach single-segment PELD. The inclusion criteria were an age of 35–60 years, a diagnosis of LDH according to the clinical diagnostic criteria, and clinical manifestations consistent with the imaging findings [19]. Individuals of either sex were included. The exclusion criteria were postoperative infection, a history of lumbar surgery, a severe injury or a deformity of the lower extremities, a poor physical conditions for severe heart, lung or blood system disease, a central or peripheral nervous system disorder, a venous thrombosis of the lower extremities, cognitive impairment limiting the patient’s ability to understand motor tasks caused by a mental or psychological disorder.

### Groups

Postoperatively, two rehabilitation programme were performed for patients randomly. The staged group (25 cases) underwent a lumbar-pelvic-leg kinetic chain staged rehabilitation programme. The regular group (26 cases) underwent a regular low back muscle exercise programme.

### Treatment

#### Lumbar kinetic chain staged rehabilitation programme

The rehabilitation programme was divided into three stages performed from 2 to 6, 7–12, and 13–24 weeks postoperatively, according to the characteristics of PELD recovery. This programme mainly involved lower extremity activity to prevent perioperative complications and active spine extension to reduce intervertebral disc stress in the early stage, muscle stretching to increase muscle flexibility in the middle stage, core muscle strengthening and posture and gait correcting components in the later stage. The specific staged rehabilitation programme is shown in Table 1.

**Table 1:** Staged rehabilitation programme for the staged group

Timing	Purpose	Treatment points	Notes	Basic exercises	Selective actions
1–3 days	To control postoperative pain and prevent complications	Rest in bed, turn over axially with medication. Limb active movement training, lower extremity passive joint mobility training or active joint mobility training.	Assess lower extremity sensation and motor function. Use analgesics when necessary. Patients who did show a worsened condition after the operation entered the lower-level rehabilitation.	Passive straight-leg raising exercises	Bridge support (Single bridge)
4 days-2 weeks	To control waist movement and carry out physical rehabilitation.	Active training for the lower extremities in bed, straight leg raising training and isometric contraction training for lumbar extensors. A waist girdle or lumbosacral brace was used to stand by the bed for 10 min 2–3 times a day.	Avoid violent straight leg raising. Apply analgesics when necessary. The patients who can complete this level of training can enter the lower level of rehabilitation.	Bridge support (Single bridge/Double bridge)	Horizontal arch movement and supine curly hug exercise
2–3 weeks	To appropriately increase the range of lumbar motion.	A hard waist or lumbosacral brace was used for indoor walking, and the walking distance was gradually increased. Equipment was selected mainly to enhance the strength of lower extremity muscles, and the intensity of the exercises was moderate.	The patients without complications were discharged. Analgesics were not used.	Bridge support Supine arch motion Supine curly hug exercise	Prone upper-limb-support stretching exercise
4–6 weeks	To gradually strengthen the lower back muscles and abdominal muscles. To gradually transition to daily activities.	With protection of the waist, gradually resume walking in the community, participate in daily life activities and non-physical work, and increase the walking distance. Selective training to increase lower extremity muscle endurance.	Replace the soft waist support. Avoid weight-bearing on the waist and straight-leg bending activities, learn to sit correctly and maintain lordosis.	Bridge support Supine arch motion Supine curly hug exercise Prone upper limb support and stretching exercise	forward bending Supine leg lift
6–12 weeks	To complete daily activities independently and allow patients to perform general work	Gradually participate in general work with waist protection. Gradually perform strength training for the core muscle groups of the spine.	Avoid physical labour and weight-bearing and follow the rehabilitation training plan.	Supine arch motion Supine curly hug exercise Prone upper limb support and stretching exercises	Sit forward and bend your legs Modified Yanfei exercise in the supine position pelvic alternate pronation exercise
12–24 weeks	To allow patients to independently complete daily activities and participate in normal work	Remove waist circumference for functional exercise of lower back gluteal muscles and participate in general work. Perform strengthening training for the core muscles of the spine.	Avoid strenuous exercise and high-intensity weight-bearing.	Prone upper-limb-support and stretching exercises Supine leg lift Modified Yanfei exercise performance Lateral pelvic strengthening exercise Crawling training	

After discharge, all patients returned to the clinic once a week to receive instructions from the therapist. Additionally, all patients received a standard informational booklet on the staged rehabilitation programme. Lumbar and hip muscle training was performed with bridging exercises, supine arch movement, and supine curly hug. Each exercise was held for 5 s and then repeated. Abdominal muscle training was performed with plank training and supine leg lift exercises. Pelvis and lower extremity training was performed with alternating pronation movements of the pelvis in the supine position, pelvic lateral strengthening exercises, and crawling training. The spine was protected, and the programme was adjusted according to each patient’s level of dysfunction, back pain and/or radiation pain.

#### Regular low back muscle exercise programme

Regular lumbar postoperative rehabilitation focused on low back muscle exercises that progressed gradually, without consideration of the specific recovery stages [5–7]. After discharge, the patients were followed up and received instructions from the therapist at 6, 12, and 24 weeks postoperatively. The programme involved the movements mimicking a flying swallow and bridge exercises. The programme was adjusted based on the feedback of the patients.

### Outcome measures

Rehabilitation assessment were performed by a trained doctor and a senior physical therapist blinded to the grouping at baseline and 6, 12, and 24 weeks postoperatively. The efficacy of the different programmes was evaluated by the low back pain visual analogue scale (VAS), Japanese Orthopaedic Association (JOA) score, Oswestry dysfunction index (ODI), simplified Chinese version of the SF-36 health survey (40–850 points), cross-sectional area (CSA) of the lumbar multifidus on MRI, and the ratio of the left to the right supporting phase of gait.

**Fig. 1:**
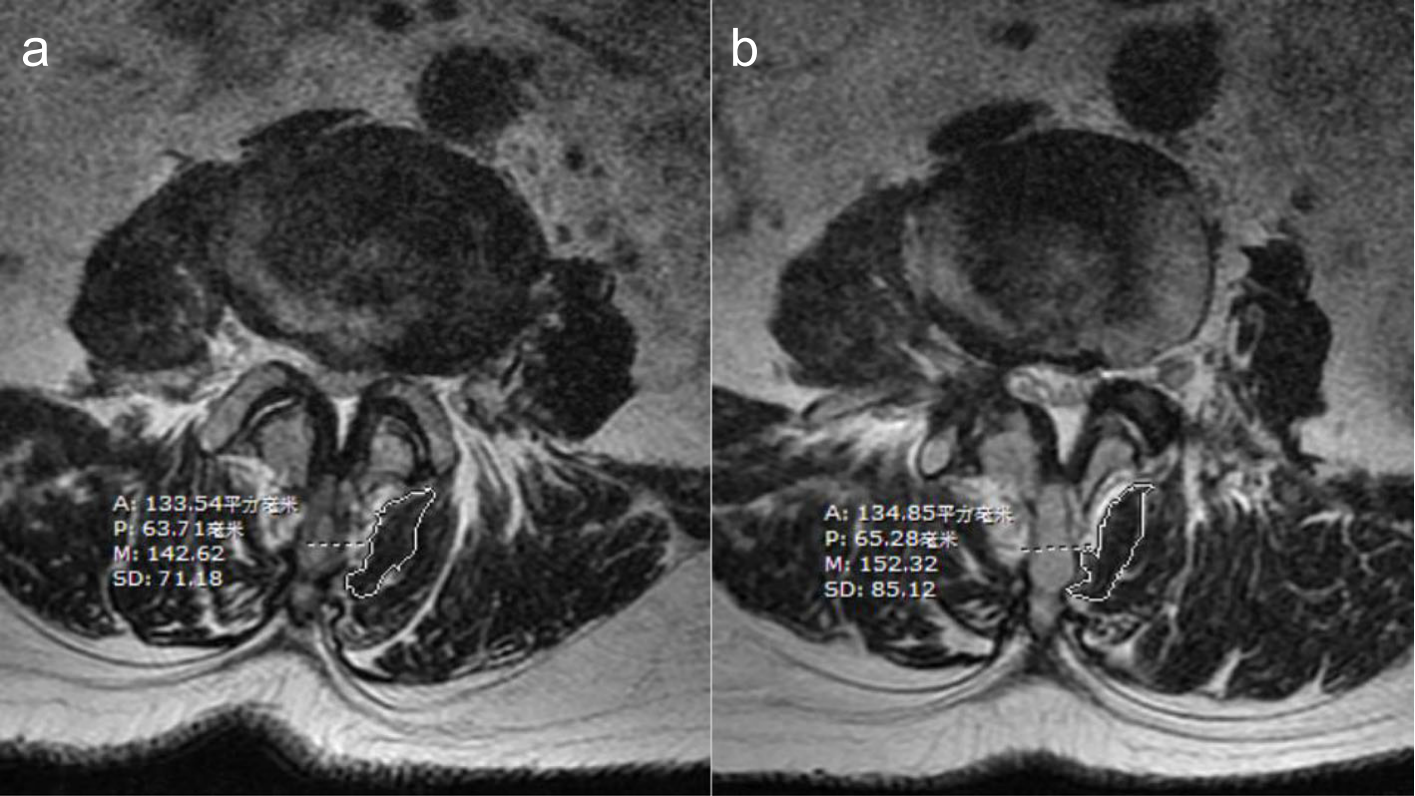
Calculation method of the CSA of the lumbar multifidus muscle on MRI. Note: The Philips MRI machine uses T2-weighted phase imaging of the L4-5 axial intervertebral space level of the lumbar spine surgery segment to select the cross-sectional boundary of the multifidus muscle by freehand tracing, and the system generates area values for area calculations. **a** (left) Patient, female, 58 years old, L4-5 intervertebral disc herniation, preoperative L4-5 disc level multifidus muscle CSA tracing method and area value (133.54 mm^2^). **b** (right) The method and area value (134.85mm^2^) of the CSA of the multifidus muscle in the L4-5 disc 6 months after surgery. A represents the CSA of the multifidus muscle (mm^2^); P indicates the perimeter of the scribed area (mm); M indicates the average gray value of the scribed area; SD indicates the standard deviation of the gray value of the scribed area

The CSA of the lumbar multifidus muscle was determined with a Philips MRI machine. On the axial view of the T2-weighted images at the level of surgical intervertebral space [14, 20], the freehand method was used to select the multifidus cross-sectional boundary and calculate its area (Fig. 1).

Gait analysis was performed with a MyoMotion 3D motion capture and analysis system. A 3D model of the human body with sensors was established. The movement angles, acceleration and movement directions of the joints were quickly obtained. The left-right support ratio was used to evaluate the symmetry of gait in this study. A ratio of 1 indicates ideal symmetry.

### Statistical analysis

Statistical analysis was performed with SPSS for Windows (version 21.0). The descriptive statistics reported are the mean and standard deviation (S.D.). The baseline characteristics were evaluated by the chi-square test for sex and the independent-samples t test for age. Intergroup and intragroup differences in the measurement parameters were evaluated by the paired-samples t test and independent-samples t test, and Levene’s Test for the equality of variances was used with the independent-samples t test. *P* < 0.05 was considered statistically significant.

## Results

In total, 51 LDH patients were included according to the inclusion criteria after PELD, and 25 patients in staged group and 26 patients in regular group completed the rehabilitation programme in the Chinese Rehabilitation Research Center from January 2019 to June 2020. No adverse events occurred in this study, such as tumbling or muscle sprains. There was no statistically significant difference in sex or age between two groups (*p* > 0.05). The baseline characteristics of the study population are shown in Tables 2 and 3.

**Table 2:** General information of the two groups

Group	Staged group	Regular group	t/✘^2^	***P***
male	13	14	0.028*	0.867
female	12	12		
Age (years)	47.9±17.2	52.2±13.7	-0.993	0.366

*indicate the results of ✘^2^

**Table 3:** Details of lumbar disc herniation in the two groups

Classification of lumbar disc herniation	Staged group	Regular group
L3-4	0	1
L4-5	13	11
L5S1	12	14
extrusion	21	20
prolapse	4	6
central	10	8
para-central	12	14
lateral	3	4

The follow-up results of the two groups were compared with the baseline results (intragroup comparisons). The VAS score decreased and the JOA and SF-36 scores increased in both groups at 6 weeks (*P* < 0.05). The ODI score of the regular group at 6 weeks and the CSA of the lumbar multifidus muscle on MRI in both groups at 12 weeks did not change significantly (*P* > 0.05) (Tables 4 and 5).

**Table 4:** Assessment results for the different stages of rehabilitation in the two groups

Item	Staged group	Regular group	t value	*P*
**pre-Reh**				
** VAS**	2.84 ± 0.69	3.00 ± 0.75	--0.794	0.431
** JOA**	17.28 ± 1.59	17.81 ± 1.52	-1.208	0.233
** Oswestry**	21.56 ± 5.27	23.73.90 ± 5.23	-1.477	0.146
** SF-36**	510.08 ± 59.71	523.27 ± 57.38	-0.804	0.425
**6 weeks post-Reh (1st stage)**				
** VAS**	1.96 ± 0.68^a^	2.42 ± 0.78^a^	-2.300	0.026
** JOA**	20.84 ± 2.19^a^	19.61 ± 1.88^a^	2.145	0.037
** Oswestry**	18.28 ± 4.70^a^	21.46 ± 4.82	-2.385	0.021
** SF-36**	633.52 ± 52.41^a^	598.31 ± 67.64^a^	2.072	0.044
**12 weeks post-Reh (2nd stage)**				
** VAS**	1.08 ± 0.40^a^	1.38 ± 0.70^a^	-1.903	0.063
** JOA**	23.88 ± 2.43^a^	22.88 ± 2.86^a^	1.335	0.188
** Oswestry**	14.68 ± 2.86^a^	17.38 ± 3.15^a^	-3.202	0.002
** SF-36**	721.52 ± 55.74^a^	668.11 ± 71.38^a^	2.970	0.005
**24 weeks post-Reh (3rd stage)**				
** VAS**	0.48 ± 0.51^a^	0.69 ± 0.55^a^	-1.429	0.159
** JOA**	27.88 ± 1.17^a^	27.46 ± 1.21^a^	1.258	0.214
** Oswestry**	11.48 ± 1.26^a^	11.38 ± 0.85^a^	0.317	0.752
** SF-36**	775.80 ± 55.48^a^	746.54 ± 40.69^a^	2.154	0.036

Note: compared to pre-Reh

^a^*p* < 0.05

**Table 5:** Comparison of the cross-sectional area of the multifidus muscle and gait analysis results for both groups before and after rehabilitation

Items	Staged group	Regular group	t value	*P*
CSA of MF Pre-Reh	274.40 ± 127.67	301.35 ± 128.16	-0.752	0.456
left-right support ratio of gait analysis in Pre-Reh	0.82 ± 0.04	0.83 ± 0.02	-0.905	0.370
CSA of MF 12 weeks post-Reh	288.84 ± 128.65	316.50 ± 124.55	-0.780	0.439
left-right support ratio of gait analysis in 24 week s post-Reh	0.95 ± 0.02^a^	0.93 ± 0.02^a^	3.713	0.001

CSA of MF, cross sectional area of multifidus muscle

^a^compared to pre-Reh *P* < 0.05

There were no significant differences in the measurement parameters between the two groups before the rehabilitation programme (*p* > 0.05). The follow-up results were compared between the two groups (intergroup comparisons) at different rehabilitation stages. In the staged group, compared with the regular group, the VAS and ODI scores were lower and the JOA and SF-36 scores were higher at 6 weeks (*P* < 0.05); the VAS and ODI scores were lower and the SF-36 score was higher at 12 weeks (*P* < 0.05); the SF-36 score was higher at 24 weeks (*P* < 0.05); the CSA of the lumbar multifidus showed no differences at 12 weeks (*P* > 0.05); and the ratio of the left to the right supporting phase of gait was higher at 24 weeks (*P* < 0.05) (Tables 4 and 5).

## Discussion

Because the main problem varies across recovery stages after PELD, this study developed a staged rehabilitation programme that differed by the period after surgery. The results indicated that the patients in the staged group exhibited significantly improved low back pain and lumbar spine function in 6th and 12th weeks, and the programme promoted patient recovery from the surgery. At 12th week, the left-right support phase ratio of gait was significantly higher in the staged group than in the regular group; at 24th week, the results of the low back pain and lumbar spine function evaluation were similar between the two groups, but the staged group was relatively healthier, and the CSA of the multifidus muscle in the surgical segment was nonsignficantly larger, showing that the application of the staged rehabilitation programme yielded an efficient clinical effect.

Functional units of the spine (passive system), paravertebral muscle system (active system) and neuromuscular control system (regulatory system) work together to execute spine-related movements. Coordinated control of the human spine and extremity kinetic chain is the key to normal motor function [21, 22]. The theory of spine rehabilitation, which emphasizes the overall movement of the spine, is reasonably suitable for postoperative rehabilitation to improve surgical efficacy, reduce postoperative complications and the severity of pain, maximally restore patients’ physical function [23].

McKenzie therapy is a widely recognized nonsurgical treatment for low back pain [18, 24], especially by physical therapists. In some countries, this therapy is designated as a standardized rehabilitation programme for chronic low back pain [25, 26]. The basic principles of McKenzie therapy for low back pain are to return the joints of the spine to the neutral position through special posture training and exercise and to return the disc to the central position of the intervertebral space, avoiding movements or postures that push the intervertebral disc toward the edge [18, 27]. In theory, this treatment can prevent the recurrence of LDH in the surgical segment after PELD and the degeneration of the adjacent segment, biomechanically aligning the spine with the human body.

The core of the human body is defined as the lumbar-pelvic-hip complex, which is where all movement in the body begins, and 29 muscles are connected to this complex [23]. Effective core strength facilitates optimal kinematic performance of the functional motor chain, providing neuromuscular efficiency throughout the chain and proximal stability during lower extremity movement. Good human core strength also provides normal dynamic stability to generate forces and counteract abnormal stresses. When the nucleus pulposus is removed, it is likely that lumbar facet joints and the ligamentum flavum are partly excised during PELD, which inevitably leads to altered kinematics of the overall kinetic chain and a reduced ability to counter abnormal external forces. Therefore, maintaining kinetic chain coordination as much as possible through core muscle strengthening exercises is an important remedy and the most effective type of functional exercise. Several studies have clearly demonstrated that core stability training can improve the symptoms of chronic low back pain and prevent the recurrence of low back pain [28–30]. The results of this study confirmed that the CSA of the surgical segmental multifidus muscle increases at 24 weeks of staged rehabilitation; although the change was not statistically significant, it helped to improve the core stability of the body.

Patients with LDH often present with pelvic kinetic dysfunction, which causes gait abnormalities [15, 16]. Pelvic kinematics and lumbopelvic rhythms are used to evaluate the mechanisms by which LDH causes gait abnormalities. Changes in pelvic posture occur with anterior, posterior, coronal tilt and horizontal rotation of the lumbar spine and hip joint. Patients with herniated discs experience anterior pelvic tilt to prevent the nucleus pulposus from moving backward. The lumbopelvic rhythm responds to the sagittal flexion of the lumbar spine and hip joints during trunk movement from upright to flexed, and vice versa. Patients with LDH exhibit compensatory pelvic motion due to abnormal lumbar curvatures or pain and thus eventually exhibit gait abnormalities. This study showed abnormalities in the bilateral leg support ratio during gait, and after 12 weeks of staged rehabilitation, this gait abnormality was effectively corrected. This finding confirms that pelvic lower extremity dysfunction after LDH is of great significance.

Early rehabilitation after PELD is mainly conducted to promote healing of the tissues around the surgical site, enable standing as early as possible with waist support, and prevent early complications, such as venous thrombosis of the lower extremities. In this study, both groups underwent the same type of rehabilitation for 2 weeks postoperatively, which was in accordance with the medical ethics guidelines. When the patients were discharged from the hospital, the two groups began to undergo strict staged rehabilitation or regular rehabilitation, respectively. Significant improvements in low back pain, lumbar spine function and ability to perform activities of daily living were found in the staged group at the 6th and 12th weeks postoperatively. Early and targeted postoperative rehabilitation improves surgical efficacy, improves symptoms, and promotes the recovery of physical function. At 24th weeks postoperatively, there was improvement in pain and lumbar function in both groups, but only the SF-36 score in the staged group improved significantly. Both groups of patients underwent rehabilitation exercises, and after 6 months of rehabilitation, the patients recovered from surgical trauma, which allowed the body to reorganize its structure and function in daily life. Staged rehabilitation was targeted more, with more systematic exercises of the lumbar, abdominal, and back muscles, and patients exhibited better recovery of core strength, greater adaptability and better SF-36 scores. Thus, staged rehabilitation is important not only to promote symptomatic improvement and functional recovery after surgery but also to help patients relearn proper movement patterns. There were even some patients who exhibited higher levels of athletic performance after staged rehabilitation than before surgery.

Previous studies [31] have suggested the lumbar kinetic chain training after PELD. In this study, staged rehabilitation was specially designed according to the characteristics of recovery after PELD, which hope to increase the safety and rationality of rehabilitation programme after PELD. In this preliminary study, only a sample size of 51 patients were included. In the future, multicentre randomized controlled studies with larger sample sizes should be conducted. Carrying out staged rehabilitation and medium- and long-term follow-ups in the community or at home would improve the cost-effectiveness of the programme. Although rehabilitation protocols are consistent in same group and instructors received unified training, there may be differences in understanding and implementation by patients. The only gait analysis parameter was the ratio of the left to the right supporting phase, and muscle mechanics of the lower extremities were not assessed. The core muscle strength of the lumbar region should be evaluated in future studies.

## Conclusions

The staged rehabilitation programme proposed in this study showed higher efficacy than did regular low back muscle exercise during rehabilitation after PELD for LDH. A specific staged rehabilitation programme and lumbar kinetic chain training are helpful for improving the clinical effect of PELD.

## Data Availability

The datasets used and/or analyzed during the current study are available from the corresponding author on reasonable request.

## Abbreviations

LDH: Lumbar disc herniation
PELD: Percutaneous endoscopic lumbar discectomy
VAS: Visual analogue scale
JOA: Japanese Orthopaedic Association score
ODI: Oswestry dysfunction index
SF-36: Simplified Chinese version of the health survey (40-850 points)
CSA: Cross-sectional area
S.D.: Standard deviation
MF: Multifidus muscle
